# Mechanical Properties and Cytocompatibility Improvement of Vertebroplasty PMMA Bone Cements by Incorporating Mineralized Collagen

**DOI:** 10.3390/ma8052616

**Published:** 2015-05-13

**Authors:** Hong-Jiang Jiang, Jin Xu, Zhi-Ye Qiu, Xin-Long Ma, Zi-Qiang Zhang, Xun-Xiang Tan, Yun Cui, Fu-Zhai Cui

**Affiliations:** 1 Wendeng Orthopaedic Hospital, No. 1 Fengshan Road, Wendeng 264400, Shandong, China; E-Mails: boneman@163.com (H.-J.J.); jboneman@sina.com (X.-X.T.); 2 Kangda College of Nanjing Medical University, No. 8 Chunhui Road, Xinhai District, Lianyungang 222000, Jiangsu, China; E-Mail: xujin33@hotmail.com; 3 School of Materials Science and Engineering, Tsinghua University, Haidian District, Beijing 100084, China; E-Mail: ye841215@gmail.com; 4 Beijing Allgens Medical Science and Technology Co., Ltd., No. 1 Disheng East Road, Yizhuang Economic and Technological Development Zone, Beijing 100176, China; E-Mails: zhangzq@allgensmed.com (Z.-Q.Z.); cuiyun@allgensmed.com (Y.C.); 5 Tianjin Hospital, No. 406 Jiefang South Road, Tianjin 300211, China; E-Mail: maxinlong8686@sina.com

**Keywords:** mineralized collagen, polymethyl methacrylate bone cement, vertebroplasty, compressive elastic modulus, cytocompatibility

## Abstract

Polymethyl methacrylate (PMMA) bone cement is a commonly used bone adhesive and filling material in percutaneous vertebroplasty and percutaneous kyphoplasty surgeries. However, PMMA bone cements have been reported to cause some severe complications, such as secondary fracture of adjacent vertebral bodies, and loosening or even dislodgement of the set PMMA bone cement, due to the over-high elastic modulus and poor osteointegration ability of the PMMA. In this study, mineralized collagen (MC) with biomimetic microstructure and good osteogenic activity was added to commercially available PMMA bone cement products, in order to improve both the mechanical properties and the cytocompatibility. As the compressive strength of the modified bone cements remained well, the compressive elastic modulus could be significantly down-regulated by the MC, so as to reduce the pressure on the adjacent vertebral bodies. Meanwhile, the adhesion and proliferation of pre-osteoblasts on the modified bone cements were improved compared with cells on those unmodified, such result is beneficial for a good osteointegration formation between the bone cement and the host bone tissue in clinical applications. Moreover, the modification of the PMMA bone cements by adding MC did not significantly influence the injectability and processing times of the cement.

## 1. Introduction

Vertebral compression fractures (VCF) are one of the most common fractures for the elders with osteoporosis. In the United States, it was reported that about 25% of postmenopausal women suffered from VCF, and such morbidity rate was estimated to be 40% for those women over 80 years old [1]. With the current accelerated trend of the aging of the world population, the occurrence of VCF will continue increasing. Besides osteoporosis, VCF can also be induced by other disease, such as osteogenesis imperfecta [2], spinal tumors [3], and so on.

Percutaneous vertebroplasty (PVP) and percutaneous kyphoplasty (PKP) are the major applications of the polymethyl methacrylate (PMMA) bone cement in the treatment of VCF. In either PVP or PKP, the bone cement is injected into the vertebral body for the augmentation of the fractured vertebral body. The immediate effect and safety of the PMMA bone cements used in PVP and PKP have been deeply investigated and verified by long-term clinical practices. However, existing commercially available PMMA bone cement products for PVP and PKP have been reported to cause some complications, mainly includes secondary fractures of the adjacent vertebral bodies, and loosening or even dislodgement of the set PMMA bone cement, due to the high elastic modulus and bioinert of the PMMA.

The compressive elastic modulus of normal human vertebral body is 50–800 MPa [4,5,6], while the PMMA bone cements form hard solid body with an elastic modulus of 2000–3700 MPa [7,8], which is much higher than that of normal human vertebral body. The vertebral body filled with PMMA bone cement has a significantly higher stiffness than the adjacent segments, and the resulting stress concentration will easily cause secondary fracture on adjacent vertebral bodies and endplate near the surgical segment [9,10]. The incidence of the secondary fracture of the adjacent bodies after PVP and PKP was reported as high as 7%–20% [11], which is 4.62 times than those occurred on other segments [12].

On the other hand, PMMA is a bioinert material that neither form chemical bonding, nor form osteointegration with the bone tissue at the implant site [13], resulting in obvious interface and weak combination strength between the bone cement and the host bone. Micro motion cannot be avoided under such weak combination in daily activities, and small wear debris produced by the micro motion would cause osteolysis and further aseptic loosening or even dislodgement of the bone cement implant [14,15].

A new PVP or PKP surgery, or even more are necessary for the treatment of the secondary fracture on the adjacent vertebral body, which increase pain and economic burden of the patient. For serious loosening or dislodgement of the bone cement, further revision surgery is inevitable. Therefore, the modification of PMMA bone cement for the treatment of VCF is important and extremely urgent for clinical applications. Many approaches were tried to improve mechanical properties and/or biocompatibility of the PMMA bone cement by, for example, adding biocompatible hydroxyapatite (HA) powder, or partially modifying methyl methacrylate (MMA) monomer. However, ideal results were not achieved by previous reported modification studies, since the compressive strength decreased too much to meet the requirement of corresponding standard (ISO 5833-2002), or the compressive elastic modulus increased rather than decreased, or the injectability was limited and is not available in the use of PVP or PKP.

Mineralized collagen (MC) is a biomimetic biomaterial with the same chemical composition and hierarchical structures to natural bone tissue. The MC is usually prepared by an *in vitro* biomimetic mineralization process that is similar to the formation of natural bone tissue [16,17]. Within the MC, the organic type-I collagen is orderly arranged with the inorganic nano-sized HA [16]. Many laboratory studies and clinical practices have demonstrated that the MC could be used to fill bone defects and is able to promote new bone formation at the bone defect sites [18,19].

In this study, MC particles were added to commercially available PMMA bone cement products to improve both the mechanical properties and the cytocompatibility. The modification parameters, including MC particle size range and additive percentage were investigated for each PMMA bone cement. Injectability, mechanical properties, maximum temperature and setting time were tested to determine the modification availability and effectiveness. Cell experiments were performed to evaluate cytocompatibility improvement of the modification by observing adhesion and quantifying proliferation of pre-osteoblasts on the modified bone cements.

## 2. Materials and Methods

### 2.1. PMMA Bone Cement Products

Three commercially available PMMA bone cement products for PVP and PKP were purchased. The three products were Osteopal^®^ V (Heraeus Medical GmbH, Hanau, Germany), Mendec^®^ Spine (Tecres S. P. A., Verona, Italy) and Spineplex™ (Stryker Instruments, Kalamazoo, MI, USA). All these three bone cements were certified by medical administration of many countries and regions, and have been used in clinics for many years.

### 2.2. Preparation of MC Particles

MC particles used for the modification of the PMMA bone cements were made from a commercially available artificial bone graft “BonGold” produced by Beijing Allgens Medical Science and Technology Co., Ltd. (Beijing, China). The MC bone grafts were prepared by following main steps described in [20]. Briefly, water-soluble calcium salt solution and phosphate salt solution were added into acidic collagen solution to form MC deposition by adjusting pH value and temperature of the reaction system. This step is a biomineralization process, which was similar to the mineralization process of the natural bone tissue that the HA crystal nucleation and growth were directed by collagen molecular templates. The deposition was then collected by centrifugation and freeze-dried to obtain MC bone graft product.

The MC bone graft was ground into small particles and screened out 4 groups with different particle sizes by sieving. The particle size range for each group was: <200 μm, 200–300 μm, 300–400 μm, and 400–500 μm, respectively. Since the inner diameter of bone filler device for delivering bone cement in PVP and PKP are usually 2.5–4.0 mm, MC particles less than 500 μm were used in this modification study.

### 2.3. Addition Methods of the MC

MC particles with different addition amounts and size ranges were added into the bone cements for the modification. In our preliminary experiments, too much MC addition (>20wt % of the powder part of the bone cement) would lead to hard stirring of the bone cement and losing injectability. Therefore, 4 addition amount groups, 5 wt%, 10 wt%, 15 wt%, and 20 wt% of the powder part of the bone cement, were studied for each particle size range.

In the modification process, powder and liquid parts of the bone cement were firstly mixed for 30 s to form a uniform flowing phase, and MC particles were then added into with rapid stirring for 30 s to ensure homogeneous distribution within the bone cement. There were two adding methods for the MC particles. One is direct addition of a certain amount of MC particles, the other is partial replacement of the powder part of the bone cement by equivalent amount of MC particles. Specifically, in the replacement method, a portion of the powder part of the bone cement was firstly removed, and then the MC particles equivalent to the removed bone cement powder in weight would be added into the mixed bone cement. The direct addition is preferred since such operation is convenient for clinical use.

### 2.4. Injectability of the Modified Bone Cements

A bone filler device with an inner diameter of 2.8 mm (Shanghai Kinetic Co., Ltd., Shanghai, China) was used to investigate the injectability of the MC modified PMMA bone cements. The uniformly mixed bone cement was extracted into a 20 mL syringe, injected into the bone filler device, and then pushed out to determine whether the modified bone cement was injectable or not.

### 2.5. Mechanical Property Tests

Mechanical properties of the MC modified PMMA bone cements were tested by using a universal materials testing machine (Instron-5880, Instron, Norwood, MA, USA) according to annex E and F of ISO 5833-2002. Cylindrical specimens with 6 diameter and 12 mm height were prepared for compressive strength and compressive modulus tests, and flat plate specimens with 75 mm length, 10 mm width and 3.3 mm depth were prepared for four-point bending strength and bending modulus tests.

The compressive strength, bending strength and bending modulus for each specimen were calculated according to related expressions provided by ISO 5833-2002. The compressive modulus for each specimen was calculated as the slope of the linear region of the stress-strain curve, which was derived from the displacement-load curve recorded by the testing machine, the height and the diameter of the specimen.

### 2.6. Maximum Temperature and Setting Time Tests

Maximum temperature and setting time of the MC modified PMMA bone cement were tested and recorded as described by annex C of ISO 5833-2002. Briefly, approximately 25 g immediately mixed bone cement was filled into a polytetrafluoroethylene (PTFE) mold, and the temperature was measured via a thermocouple and an electronic converting device having an accuracy of ±0.1 °C. The maximum temperature would be directly recorded by the electronic converting device, and the setting time was determined as the time corresponding to the average value of the maximum and the ambient temperature [21]. The best modification solution screened by above mechanical property tests was tested for each PMMA bone cement product, and the two parameters of each unmodified original product were also tested as the control. The tested were performed at 23 °C and relative humidity of 50%.

### 2.7. Processing Times Tests 

Processing times are of importance for clinical operation of the bone cements by a surgeon. The processing times consisted of four phases, including mixing, waiting, application, and setting. In this study, processing times were tested for each bone cement before and after the modification to investigate the influence of the MC addition on the operation properties of the bone cements.

The measurement principles for the four phases were as follows:

Mixing time: time for completely mixing of the powder part and liquid part of the bone cement, as well as the MC particles;
Waiting time: time from the bone cement being extracted in to the syringe to being suitable for the injection;
Application time: time from the bone cement being applicable to being hard to inject;
Setting time: time from the injection of the bone cement to it become hardened.

### 2.8. In Vitro Cytocompatibility Evaluation

Cytocompatibility improvement of the MC modified bone cement were evaluated by culturing pre-osteoblasts on modified and unmodified Osteopal^®^ V and Mendec^®^ Spine bone cements. The use of these two bone cements was because that they contained different contrast agents, Osteopal^®^ V contained ZrO_2_ and Mendec^®^ Spine contained BaSO_4_ in their respect powder part. A clonal osteogenic cell line derived from newborn mouse calvarias, MC3T3-E1 (purchased from Cell Bank of Chinese Academy of Sciences, Shanghai, China), was used in this cytocompatibility evaluation. The cells were cultured in Dulbecco’s Modified Eagle Medium (DMEM) with 10% fetal bovine serum (FBS), 100 U/mL penicillin and 0.1 mg/mL streptomycin at 37 °C in an incubator with 5% CO_2_.

To prepare bone cement samples for cell culturing, the modified and unmodified bone cements were injected into respective 5 wells in a 96-well plate with 0.1 mL per well, immediately after all the components were fully mixed together. After setting for 24 h, cells were seeded on the set bone cements by adding 100 μL cell suspension into each well at a concentration of 1 × 10^5^ cells/mL. Wells without bone cement were seeded with cells as the control group. Four such 96-well plates were maintained at 37 °C in an incubator with 5% CO_2_, and the culture medium was replaced by fresh medium 1, 3, 5 and 7 days after the cell seeding.

Cell proliferation on both modified and unmodified PMMA bone cements were tested by cell counting kit-8 (CCK-8, Dojindo, Japan) at the 1st, 3rd, 5th and 7th day after cell seeding. At each time point, one 96-well plate was randomly selected after refreshing culturing medium, and 10 μL of CCK-8 solution was added into each well. After 2 h incubation at 37 °C, 100 μL solution of each well was transferred to another 96-well plate. Optical density (OD) values at 450 nm of all the wells were measured by a microplate reader (Bio-Rad, Model 680, Hercules, CA, USA).

Cytocompatibility improvement of the modified bone cement was also studied by observing cell attachment on the bone cements before and after the modification. The bone cement samples used for SEM observation of cell attachment were discs with 10 mm diameter and 2 mm thickness. The cell attachment was observed by scanning electron microscopy (SEM; FEI Quanta 200, Hillsboro, OR, USA) 48 h after cell seeding. Samples for the SEM observation were prepared as follows: bone cement samples with the cells were washed with phosphate buffer saline (PBS) to remove any non-adherent cells, and fixed in 2.5% glutaraldehyde in PBS for 24 h; the samples were then dehydrated in ascending series of ethanol solution from 50% to 100% and stored in frozen tert-butyl alcohol (TBA); followed by thoroughly freeze-drying, cell samples were sputter-coated with nano gold particles and observed by SEM.

### 2.9. Statistical Methods

The results were compared using standard analysis of Student’s *t*-test and expressed as means ± SD. *p* < 0.05 was considered statistically significant.

## 3. Results

### 3.1. Injectability of the Modified Bone Cements

Table 1, Table 2 and Table 3 list the injectability of the MC modified bone cements. The symbol “○” refers to injectable, and “×” refers to uninjectable. The expression “100/x” means the direct addition method, and “(100 − x)/x” means the replacement method.

**Table 1:** The injectability of the MC modified Osteopal^®^ V bone cement.

Particle size (μm)	Powder part of the bone cement/MC particle (w/w)								
	100/0	100/5	100/10	100/15	100/20	95/5	90/10	85/15	80/20
<200	○	○	×	×	×	○	×	×	×
200–300	○	○	×	×	×	○	○	○	×
300–400	○	○	○	×	×	○	○	○	○
400–500	○	○	○	×	×	○	○	○	×

**Table 2:** The injectability of the MC modified Mendec^®^ Spine bone cement.

Particle size (μm)	Powder part of the bone cement/MC particle (w/w)								
	100/0	100/5	100/10	100/15	100/20	95/5	90/10	85/15	80/20
<200	○	○	×	×	×	○	×	×	×
200–300	○	○	○	○	×	○	○	○	○
300–400	○	○	○	○	×	○	○	○	○
400–500	○	○	○	○	×	○	○	○	○

**Table 3:** The injectability of the MC modified Spineplex™ bone cement.

Particle size (μm)	Powder part of the bone cement/MC particle (w/w)								
	100/0	100/5	100/10	100/15	100/20	95/5	90/10	85/15	80/20
<200	○	○	×	×	×	○	×	×	×
200–300	○	○	○	×	×	○	○	○	×
300–400	○	○	○	○	×	○	○	○	×
400–500	○	○	○	○	×	○	○	○	×

The results show that either small particles or high MC addition amount largely affected the injectability of the bone cement. For Osteopal^®^ V bone cement, the equivalent placement method less influenced the injectability than the direct addition method. Once a MC modified bone cement was extracted into the syringe, it can easily be injected into the bone filler device and then be pushed out.

### 3.2. The Appearance of the Modified Bone Cements

**Figure 1:**
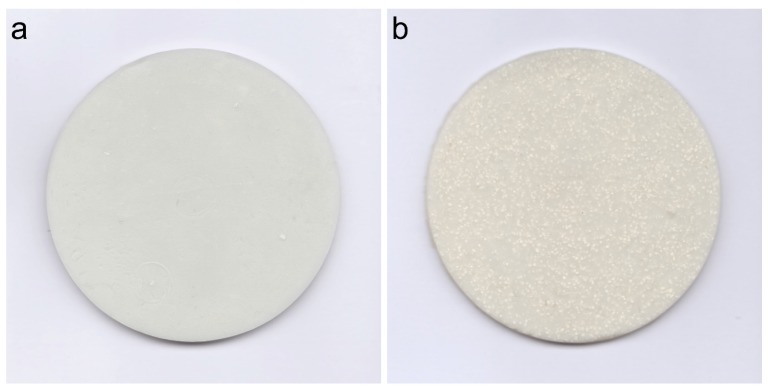
Appearance of the (**a**) unmodified PMMA bone cement and (**b**) MC modified PMMA bone cement.

Figure 1 shows the appearance of the unmodified and MC modified Spineplex™ bone cement. MC particles were homogeneously dispersed in the polymerized PMMA without obvious aggregation or vacancy, indicating that the MC particles were mixed well within the bone cement during flowing phase and MC was compatible with the PMMA material. The homogeneity of the MC modified bone cement ensures uniform mechanical properties throughout the bone cement, thus avoiding stress concentration in clinical applications.

### 3.3. Mechanical Properties of the Modified Bone Cements

#### 3.3.1. Mechanical Properties of the Modified Osteopal^®^ V Bone Cement

**Figure 2:**
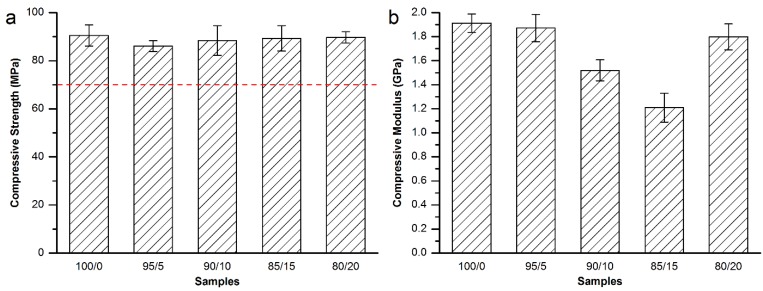
(**a**) Compressive strength and (**b**) compressive modulus of the MC modified Osteopal^®^ V bone cement.

For Osteopal^®^ V bone cement, partial replacement of the powder part of the bone cement by equivalent 400–500 μm MC particles kept injectable. Therefore, mechanical properties of different amount of 400–500 μm MC modified bone cement were tested, so as to screen out the best modification resolution. The compressive strength and modulus are shown in Figure 2.

As shown in Figure 2a, the addition of the MC particles did not affected the compressive strength of the Osteopal^®^ V bone cement. There were no significant differences between the control and each experimental group, or among experimental groups. The compressive strength for each group was higher than the 70 MPa specified by ISO 5833-2002 (red dash line in Figure 2a), thus meeting the requirement of clinical applications. Figure 2b demonstrates that replacement of the bone cement powder part by 10 wt% or 15 wt% could obtain significant down-regulation effects. 90/10 group down-regulated 20.6% and 85/15 group down-regulated 36.8%. There were statistical differences between 85/15 group and each of the other groups. Other experimental groups achieved very small down-regulation effects.

**Figure 3:**
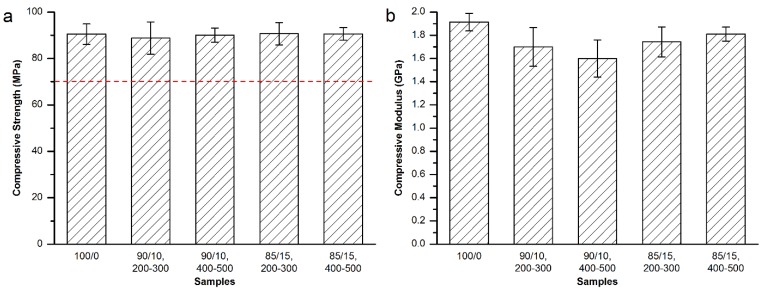
(**a**) Compressive strength and (**b**) compressive modulus of 200–300 μm and 300–400 μm MC particles modified Osteopal^®^ V.

In order to investigate the effects of the particle size range on the compressive mechanical properties of the modified bone cements, and obtain the best modification result, nearby factors and levels of above experimental groups were further tested. MC particles with 200–300 μm and 300–400 μm were used to prepared 90/10 and 85/15 groups, respectively. The compressive strength and modulus are shown in Figure 3.

As shown in Figure 3a, the addition of MC particles with the particle size of either 200–300 μm or 300–400 μm did not affect the compressive strength of the set bone cements (red dash line in Figure 3a). Figure 3b demonstrated that the replacement of the bone cement powder part by 10wt % of 400–500 μm MC particles could obtain a 16.4% down-regulation effect on the compressive modulus, which was statistically different from the control (100/0) group or (85/15, 400‒500) group. However, the modification results were much inferior to the 90/10 and 85/15 groups shown in Figure 2b. Therefore, equivalent replacement of Osteopal^®^ V bone cement powder part by 15 wt% MC particles with 300–400 μm particle size achieved the best modification result for the compressive modulus of the bone cement.

**Figure 4:**
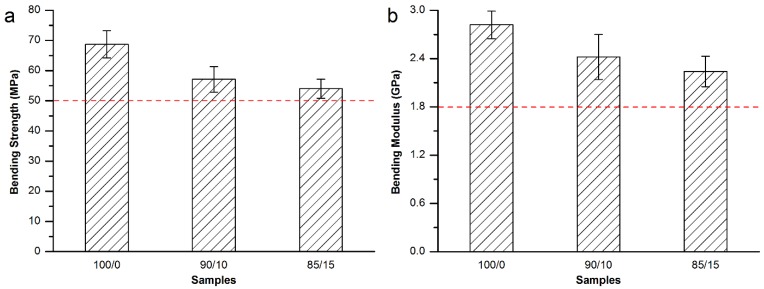
(**a**) Bending strength and (**b**) bending modulus of the MC modified Osteopal^®^ V bone cement.

Then, bending strength and modulus were tested for the Osteopal^®^ V bone cement specimens modified by equivalent replacement of the bone cement powder part by 10 wt% and 15 wt% MC particles with 300–400 μm particle size. As shown in Figure 4, it can be seen from the Figure 4 that both of the bending strength and bonding modulus decreased with the partial replacement of the powder part by MC particles. However, both of the bending strength and bending modulus were in conformity with related requirements in ISO 5833-2002 (red dash lines in Figure 4).

As a result, equivalent replacement of the bone cement powder part by 15 wt% MC particles with 300–400 μm particle size was considered to be the best resolution for the modification of the Osteopal^®^ V bone cement. The mechanical properties met the requirement of the standard and the clinical applications after the modification.

In light of the modification study on Osteopal^®^ V bone cement, 10 wt%–15 wt% were found to obtain better modification effects than other addition amounts. Moreover, the more MC contained within the bone cement, the better modification effects achieve for cytocompatibility improvement. Therefore, 15 wt% addition amount of MC particles was considered prior to other amounts, and different particle size ranges were investigated on the premise of injectability.

#### 3.3.2. Mechanical Properties of the Modified Mendec^®^ Spine Bone Cement

**Figure 5:**
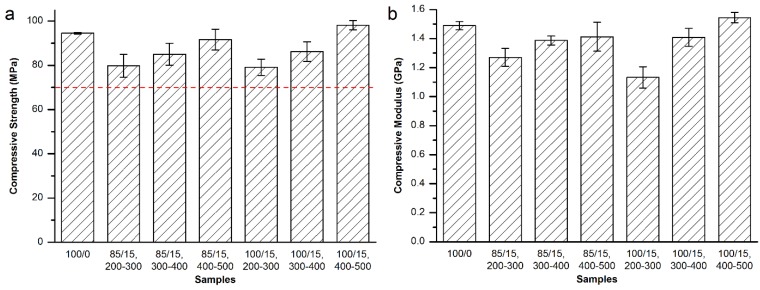
(**a**) Compressive strength and (**b**) compressive modulus of the MC modified Mendec^®^ Spine bone cement.

MC particles with the size ranges of 200–300 μm, 300–400 μm and 400–500 μm were used for the modification of Mendec^®^ Spine bone cement. The addition amount was 15 wt% for each group, and both direct addition and equivalent replacement methods were investigated. The compressive strength and modulus are shown in Figure 5.

As shown in Figure 5a, the compressive strength of the MC modified Mendec^®^ Spine bone cement met the requirement of ISO 5833-2002 (red dash line in Figure 5a). Wherein, the direct addition of 200–300 μm MC particles achieved the best effect that the compressive modulus decreased by 24.0% (Figure 5b), and was statistically different from each of the other groups. Although the equivalent replacement using the same particle size range also obtained obvious down-regulatory effect, the direct addition would be more convenient.

**Figure 6:**
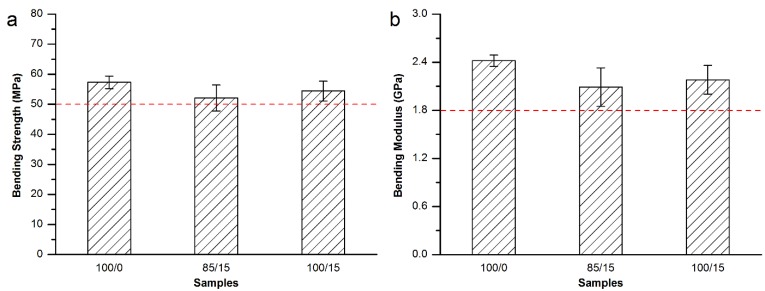
(**a**) Bending strength and (**b**) bending modulus of the MC modified Mendec^®^ Spine bone cement.

Figure 6 shows the bending strength and modulus of the Mendec^®^ Spine bone cement modified by 200–300 μm MC particles. Both specimens that modified by equivalent replacement and direct addition methods using were tested. The results show the bending strength and modulus slightly decreased by MC addition but met the requirement of ISO 5833-2002 (red dash lines in Figure 6).

As a result, direct addition of 15 wt% MC particles with 200–300 μm particle size was considered to be the best resolution for the modification of the Mendec^®^ Spine bone cement.

#### 3.3.3. Mechanical Properties of the Modified Spineplex™ Bone Cement

**Figure 7:**
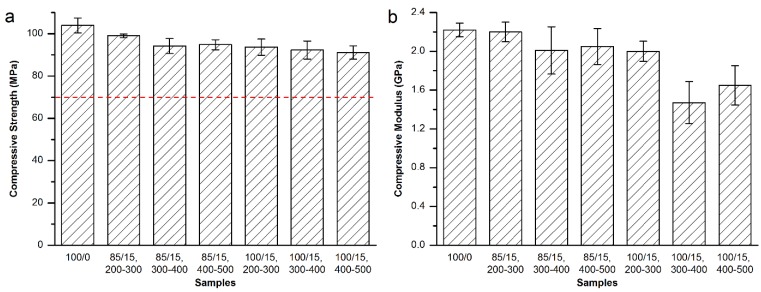
(**a**) Compressive strength and (**b**) compressive modulus of the MC modified Spineplex™ bone cement.

Similar to the study process of the Mendec^®^ Spine bone cement, six experimental groups including three particle size ranges and two addition methods were investigated. The compressive strength and modulus are shown in Figure 7.

As shown in Figure 7a, the compressive strength of the Spineplex™ bone cement modified by MC particles slightly decreased, and met the requirement of ISO 5833-2002 (red dash line in Figure 7a). Figure 7b shows direct addition of MC with 300–400 μm particle size obtained the best modification effect that the compressive modulus was down-regulated by 33.8%, and was statistically different from each of the other groups, except the (100/15, 400‒500) group, which also obtained obvious down-regulation effect in comparison with the control group.

**Figure 8:**
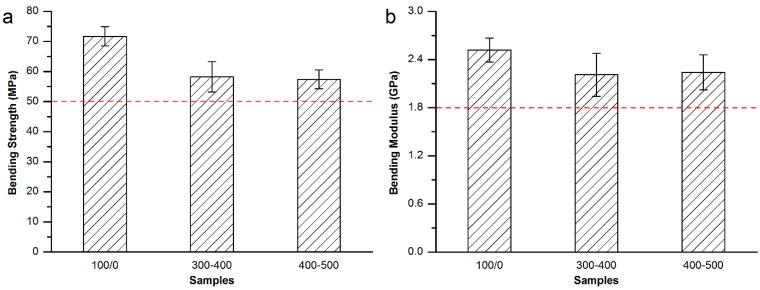
(**a**) Bending strength and (**b**) bending modulus of the MC modified Spineplex™ bone cement.

Figure 8 shows the bending strength and modulus of the Spineplex™ bone cement modified by direct addition of the MC particles. Specimens modified by 300–400 μm and 400–500 μm MC particles were tested. The results show the bending strength and modulus decreased a little after the modification but met the requirement of ISO 5833-2002 (red dash line in Figure 8).

As a result, direct addition of 15 wt% MC particles with 300–400 μm particle size was considered to be the best resolution for the modification of the Spineplex™ bone cement.

### 3.4. Maximum Temperature and Setting Time

**Figure 9:**
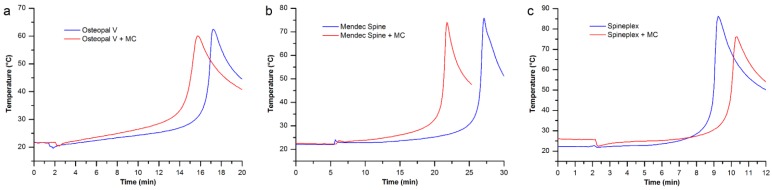
The maximum temperature comparisons between the unmodified and modified bone cements: (**a**) Osteopal^®^ V bone cement; (**b**) Mendec^®^ Spine bone cement; and (**c**) Spineplex™ bone cement.

The maximum temperature comparisons between the unmodified bone cements and their perspective optimal modification group are shown in Figure 9.

For each bone cement product investigated in this study, the maximum temperature of the modified bone cement decreased compared to its original product. Because the added MC particles absorbed a portion of heat generated by the polymerization of the PMMA bone cements. The lower maximum temperature is beneficial for clinical applications, since such low temperature could reduce damage on tissues near the bone cement caused by the heat of polymerization.

Table 4 lists the setting time of the unmodified and modified bone cements. The addition of MC particles took effects on these bone cements. Wherein, the setting time shortened for Osteopal^®^ V and Mendec^®^ Spine bone cements after the modification, while the setting time of Spineplex™ became longer. In regard to specific setting time for each bone cement, Osteopal^®^ V and Mendec^®^ Spine bone cements had overlong setting time, while Spineplex™ bone cement set too fast. A change in setting time for all bone cements, by some modification, would make it more convenient for clinical use by a surgeon.

**Table 4:** Setting time of the original bone cement products and modified bone cements.

Bone cements	Osteopal^®^ V	Mendec^®^ Spine	Spineplex™
Original product	16’44”	26’36”	9’02”
Modified by MC particles	14’51”	21’18”	10’01”

### 3.5. Processing Times for the Modified Bone Cements

**Figure 10:**
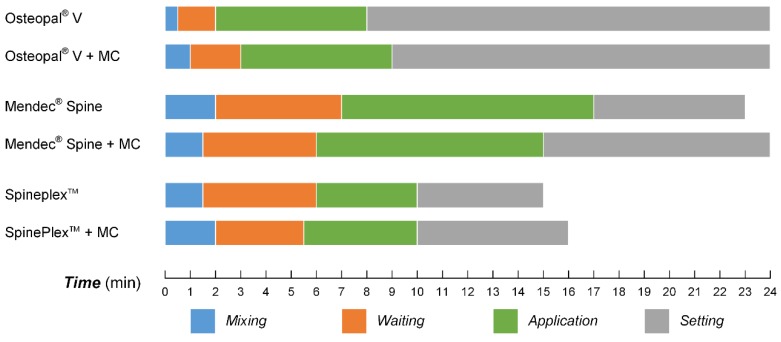
Processing times for the bone cements before and after the modification by MC.

Processing times for each bone cement, before and after the modification, are shown in Figure 10. The processing times for each bone cement varied a little after the modification by MC particles, and the variation was 0.5–1 min for each phase. Such small variation in the processing times makes no changes to the operating habits of surgeons.

**Figure 11:**
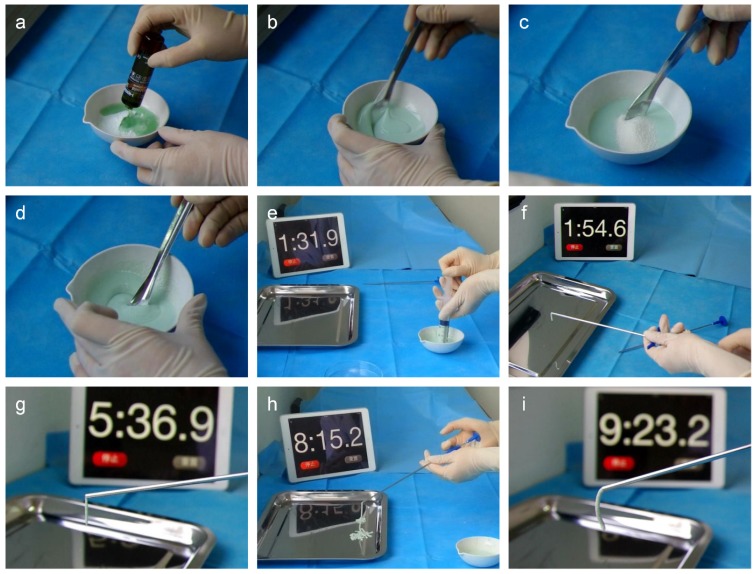
Operation process and processing times of the MC modified Osteopal^®^ V bone cement: (**a**) mixing powder and liquid parts of the bone cement; (**b**) uniformly mixing powder and liquid parts; (**c**) addition of MC particles; (**d**) uniformly mixing all components; (**e**) extracting flowing bone cement by a syringe; (**f**) injection of the bone cement into a bone filler device; (**g**) earlier stage of the bone cement; (**h**) middle stage of the bone cement; and (**i**) later stage of the bone cement.

Figure 11 takes Osteopal^®^ V bone cement as an example to show operation process and processing times of the MC modified PMMA bone cement.

### 3.6. Cytocompatibility Improvement of the Modified Bone Cements

**Figure 12:**
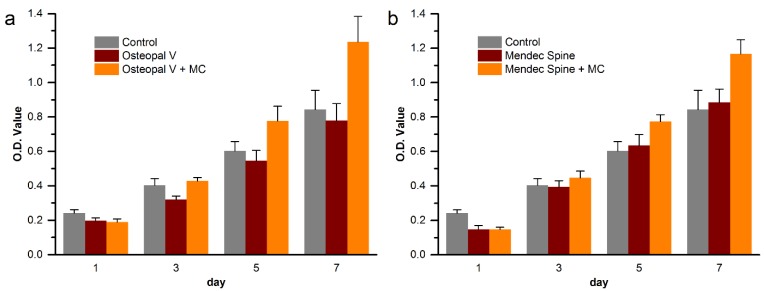
Cell proliferation on (**a**) Osteopal^®^ V and (**b**) Mendec^®^ Spine bone cements.

Cytocompatibility improvement of the MC modified bone cements were evaluated by proliferation quantification and attachment observation of MC3T3-E1 cells on the unmodified and MC modified bone cements. The proliferation of the cells on the bone cements are shown in Figure 12.

For both Osteopal^®^ V and Mendec^®^ Spine bone cements, cells proliferated well on each bone cement. Cell count on the MC modified bone cement was significantly higher than that on the unmodified original bone cement, with regard to either Osteopal^®^ V or Mendec^®^ Spine bone cement. At day 5 and 7, there were statistically significant differences between the MC incorporated group and the modified group, as well as between the MC incorporated group and the blank control. Cell counts for the unmodified group and the blank control group were closed without statistical differences at day 5 and 7, for both PMMA bone cement products, since pure PMMA bone cements and well-plates were all bioinert materials that had no effect on cell proliferation. The result indicated that the modification by using MC largely improved cytocompatibility of the PMMA bone cements, and the contrast agent, ZrO_2_ or BaSO_4_, did not affect such improvement effects.

**Figure 13:**
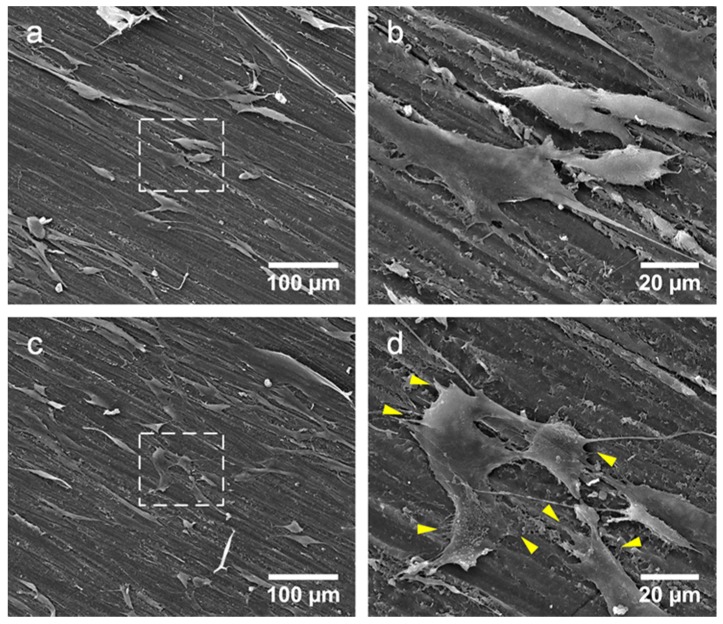
Cell observations on the bone cements before and after the MC modification by SEM: (**a**) cells on unmodified bone cement; (**b**) amplification of the center of Figure 13a; (**c**) cells on MC modified bone cement: and (**d**) amplification of the center of Figure 13c.

Figure 13 Shows cell morphology on the Osteopal^®^ V bone cement before and after the modification. Figure 13b and 13d are the amplification of the center areas (noted by dash boxes) of Figure 13a and 13c, respectively.

As shown in Figure 13, cells grew well on the bone cements and filopodia stretched out to anchor the cells on the bone cements. By comparing cells on the unmodified and modified bone cements, there were no differences on cell counts, which were in conformity with histograms shown in Figure 12. From the detail of the cell morphology shown in Figure 13b and Figure 13d, it can be seen that a large number of filopodia stretched out from the cells on the MC modified bone cement (noted by yellow arrows), while the cells on the unmodified bone cement had less filopodia. The cytocompatibility of the MC modified PMMA bone cement was better than that of the unmodified original bone cement, and cell adhesion would be improved by such modification. The results indicates that the modification of PMMA bone cements by addition of MC could improve its cytocompatibility, which is beneficial for the formation of good osteointegration between the bone cement and the host bone in clinical applications.

## 4. Discussion

The spine is the load bearing structure in the human skeleton, and vertebral bodies are the basic structural units. For upright walking human beings, the major direction of the loading on the vertebral body is compressive force in the vertical direction, including compressive force from above lower endplate and support force from bottom upper endplate. Therefore, the compressive strength and modulus are key mechanical factors for those bone cements used for PVP and PKP. Overhigh compressive modulus of the bone cement produces overhigh stiffness of the PVP or PKP treated segment, which resulting in stress concentration at the segment, and would easily cause secondary fracture on adjacent vertebral bodies and endplate near the surgical segment [9,10].

Bioinert is another disadvantage of the PMMA bone cement, since osteocytes cannot grow into the bioinert PMMA, it is unable to form stable osteointegration between the bone cement and the host bone at the implant site [13,14,15]. As described by the analysis in the introduction section, aseptic loosening or even dislodgement of the bone cement are very dangerous for patients, as a free hard block may press on the spinal nerve to produce hazardous results [22].

Many efforts were made to improve the mechanical properties and biocompatibility of the PMMA bone cements for PVP and PKP. In light of above-mentioned disadvantages, these studies were focused on down-regulation of the compressive modulus, as well as improvement of the biocompatibility of the PMMA bone cement.

As the main inorganic component of natural bone tissue, HA was popular in the modification studies on PMMA bone cements. Many studies used HA and element-doped HA, such as strontium-doped HA to modify the PMMA bone cement. However, in some studies, the addition of HA largely decreased compressive strength of the bone cement that cannot meet the requirement of ISO 5833-2002 [23]; in some other studies, the compressive modulus even largely increased after the addition of HA [24]. Moreover, the addition of HA into PMMA bone cement did not exhibit improved biocompatibility [25].

Introduction of a biodegradable component was another modification idea. For example, chitosan and sodium hyaluronate were studied to form porous structure by degradation [26,27]. However, the compressive strength of the bone cement also decreased with the degradation of the biodegradable component, and became much lower than the lower limit specified by ISO 5833-2002 [26,27].

Modification of MMA monomer was tried by some researchers to down-regulate the compressive modulus of the PMMA bone cements. For example, *N*-methyl-pyrrolidone monomer and linoleic acid were, respectively, used to partially replace the MMA monomer in the polymerization of the PMMA bone cement. However, with the down-regulation of the compressive modulus, the compressive strength also decreased to be much lower than the requirement of ISO 5833-2002 [28,29].

In summary, previous studies on the modification of PMMA bone cement did not obtain a perfect solution that both down-regulated compressive modulus without affecting the compressive strength, and improved biocompatibility of the PMMA bone cement. In this study, a biomimetic material MC with good biocompatibility and osteogenic activity was used for the modification of the PMMA bone cement. MC was compatible with the PMMA and could be homogeneously dispersed within the PMMA bone cement. The dispersed MC particles broke integrality of the polymerized bone cement, and were able to regulate mechanical properties by verifying addition amounts, particle size range, and addition method of the MC particles. Through a series of experiments, both of mechanical properties and cytocompatibilities of three commonly used PMMA bone cements for PVP and PKP were successfully improved by addition of different MC particles with different addition methods. However, related mechanical properties regulation mechanisms need further investigations, and the clinical outcomes of the modification need long-term clinical observations.

## 5. Conclusions

Biomimetic MC with good osteogenic activity was added to commercially available PMMA bone cement products to improve both the mechanical properties and the cytocompatibility in this study. As the compressive strength of the modified bone cements remained well, the compressive elastic modulus were significantly down-regulated by the MC. Meanwhile, the adhesion and proliferation of pre-osteoblasts on the modified bone cements were improved compared with cells on those unmodified. The results are beneficial for both reducing the pressure on the adjacent vertebral bodies, and the osteointegration formation between the bone cement and the host bone tissue in clinical applications. Moreover, the modification of the PMMA bone cements by adding MC did not much influence the injectability and processing times of the cement. As a result, improvement of PMMA bone cements by incorporating MC particles is an effective and easy-to-operate clinical approach for improving the quality of the surgery and reducing complications after PVP and PKP.
